# Angiogenesis and Lymphangiogenesis in Salivary Gland Adenoid Cystic Carcinoma and Mucoepidermoid Carcinoma

**DOI:** 10.31557/APJCP.2019.20.12.3547

**Published:** 2019

**Authors:** Mohammad Koochek Dezfuli, Maryam Seyedmajidi, Shima Nafarzadeh, Farzad Yazdani, Ali Bijani

**Affiliations:** 1 *Department of Oral and Maxillofacial Pathology, Mazandaran University of Medical Sciences, Sari, *; 2 *Dental Materials Research Center, Health Research Institute, *; 3 *Oral health Research Center, Health Research Institute, Dental Faculty, *; 5 *Non-Communicable Pediatrics’ Disease Research Center, Institute of Health, Babol University of Medical Sciences, Babol,*; 4 *Department of Pathology, School of Medicine, Tehran University of Medical Sciences, Tehran, Iran. *

**Keywords:** Mucoepidermoid carcinoma, adenoid cystic carcinoma, angiogenesis, lymphangiogenesis, CD31

## Abstract

**Background and Purpose:**

This study aimed to investigate the immunohistochemical expression of CD31 and podoplanin in order to examine angiogenesis and lymphangiogenesis, respectively in common malignant tumors of salivary glands.

**Materials and Methods:**

Forty formalin-fixed, paraffinated blocks (20 adenoid cystic carcinoma and 20 mucoepidermoid carcinoma blocks) were selected from the medical archives of Amir A’lam Hospital of Tehran, Iran. Sections from the blocks were stained by CD31 and D2-40 markers via immunohistochemistry. Clinical and demographic information was extracted from the patients’ records.

**Findings:**

There was a significant difference between tumors in terms of intratumoral microvessel density (MVD) (P< 0.001), total MVD (P< 0.001), and intratumoral lymphatic vessel density (LVD) (P= 0.011). In mucoepidermoid carcinoma, intratumoral MVD and LVD were greater than peritumoral MVD and LVD (P= 0.001 and P< 0.001, respectively). In mucoepidermoid carcinoma, there was no relationship between histological grade with MVD (total, intratumoral or peritumoral) or LVD (total, intratumoral or peritumoral) (P> 0.05). A similar finding was reported with respect to the histopathological grade of adenoid cystic carcinoma (P> 0.05).

**Conclusion:**

The higher level of angiogenesis and lymphangiogenesis in mucoepidermoid carcinoma, specifically at the center of tumor, compared to adenoid cystic carcinoma, may be attributed to differences in the clinical behaviors and metastasis of tumors. Moreover, considering the high LVD at the center of tumor in mucoepidermoid carcinoma and infrequency of metastasis to regional lymph nodes in adenoid cystic carcinoma, it can play a significant role in metastasis to regional lymph nodes.

## Introduction

Salivary gland tumors constitute an important part of oral and maxillofacial pathologies. Although these tumors are uncommon, they are not rare. The annual incidence of salivary gland tumors ranges from 1 to 6.5 per 10,000 people worldwide. These tumors account for 2% to 4% of all head and neck neoplasms around the world (Neville et al., 2016). They are more prevalent among adults in the fourth to seventh decade of life, and benign types are found to be more common (Kara et al., 2010). 

Mucoepidermoid carcinoma is the most common salivary gland malignancy. It commonly occurs in a wide age range from the second to seventh decade of life. In addition, it is the most common malignant salivary gland tumor in children, although it is rarely reported in the first decade of life. Some of these tumors are associated with a history of head and neck radiation. They are more commonly found in parotid glands and can be divided into three histopathological subtypes with respect to cyst formation, cellular atypia, and ratio of epidermoid, mucous, and intermediate cells. (Neville et al., 2016)

Adenoid cystic carcinoma is characterized by a combination of epithelial and myoepithelial cells, arranged in a variety of patterns: cribriform, tubular, and solid. A combination of these arrangements is generally found, and tumors are classified according to the predominant structure. The cribriform pattern is the most classic and well-known pattern (Neville et al., 2016). This type of tumor is slow-growing and infiltrative and has the tendency to invade into nerves; it is also associated with pain in patients (Gnepp, 2009). According to the literature, metastasis may extend to the lungs, bones, brain, and liver (Regezi et al., 2017), while metastasis to regional lymph nodes is uncommon (Neville et al., 2016).

Tumors require blood to grow, invade, metastasize, and supply the required oxygen and nutrients. If the tumor size exceeds 2-3 mm, angiogenesis, which is a complex process in normal and pathological conditions, is inevitable. In general, angiogenesis refers to the formation of new blood vessels from the host’s primary blood vessel structure. Since tumors have a heterozygous structure, density of blood vessels in different tumor regions is not similar (Karamysheva, 2008).

A common clinical observation suggests that carcinomas constitute metastasis through the lymphatic system, while sarcomas involve blood vessels (Kara et al., 2010). Typically, lymphatic metastasis is thought to be a passive process, during which isolated tumor cells from lymphatic vessels adjacent to the primary tumor enter lymph nodes. The entry of cancer cells into lymphatic vessels is facilitated by the lack of vessel wall integrity (Stacker et al., 2002). However, this assumption has been challenged after observing lymphatic markers and lymphangiogenic growth factors. Therefore, study of angiogenesis and lymphangiogenesis in these tumors can contribute to treatment and prognosis. Immunohistochemistry and evaluation of lymphangiogenesis and angiogenesis markers have opened new doors in this area. 

Platelet-endothelial cell adhesion molecule-1 (PECAM-1) or CD31 is a marker of angiogenesis, which expresses the hematopoietic precursor antigen, ER-MP12. It is also introduced as one of the most sensitive and useful endothelial cell markers for the assessment of angiogenesis degree in tumors (Safaei Naraghi et al., 2010). CD34, CD105, and vascular endothelial growth factor (VEGF) are also used as markers for the evaluation of angiogenesis (Ganji- Azar and Nadji, 2007). 

CD34 stains normal and neoplastic endothelial cells and has lower specificity and sensitivity than CD31 (Wick, 2008). CD105 is a significant marker for activated tumor endothelial cells of newly formed tumor vessels (Salzman et al., 2014); however, its stainability in salivary tumors is lower than that of CD31 (Fujita et al., 2011). VEGF does not have the specificity of CD31 and can also stain lymphatic vessels (Pepper, 2003). The immunohistochemical factors of lymphatic endothelial cells, such as D2-40 (podoplanin), lymphatic vessel endothelial hyaluronic acid receptor 1 (LYVE1), and VEGF receptor-3 (VEGFR-3), indicate the lymphatic vessel count (Zolfaghari Saravi et al, 2017) associated with the tumor which can metastasize to lymph nodes (Miller, 2005). 

D2-40 (podoplanin) is often used to identify lymphatic invasion and to distinguish between lymphatic and vascular invasion (Salzman et al., 2014). The significance of D2-40 marker in lymphatic endothelium is attributed to the fact that the endothelium of blood vessels is not stained with this marker (Safaei Naraghi et al., 2010). On the other hand, VEGF-R3 and LYVE1 are not reliable markers for differentiating lymphatic and blood vessels against D2-40 (Pepper et al., 2003).

With this background in mind, the aim of this study was to conduct an immunohistochemical analysis of angiogenesis and lymphangiogenesis based on CD31 and D2-40 (podoplanin) expression, respectively in common malignant salivary gland tumors, mucoepidermoid carcinoma, and adenoid cystic carcinoma.

## Materials and Methods

This cross sectional study was carried out after obtaining approval from the Research Ethics Committee of Babol University of Medical Sciences (MUBABOL.REC.1395.107). The archive of Amir A’lam Hospital of Tehran was investigated to identify patients with a diagnosis of adenoid cystic carcinoma and mucoepidermoid carcinoma. Clinical and demographic data, including age and sex, were extracted from the patients’ records. 

From the paraffinated blocks, 4 μm sections were cut and stained with hematoxylin and eosin in order to determine the degree of malignancy and histopathological subtype. A total of 40 samples (20 mucoepidermoid carcinoma and 20 adenoid cystic carcinoma samples) were confirmed after observation by a pathologist to confirm the initial diagnosis and select convenient blocks with enough tissues. The histopathological criterion was based on lesion confirmation according to the definition of tumors (Neville et al. 2016).

Primary antibodies, including CD31 and D2-40 (FLEX Monoclonal Mouse Anti-Human CD31, Endothelial Cell, Clone JC70A, RTU, Dako, Denmark; FLEX Monoclonal Mouse XH D2-40 Clone: D2-40, RTU, DAKO AS/AS+, Dako, Denmark) were used for immunohistochemical staining and heated in humidity for 30 minutes. In the next step, a post primary block solution was used to improve the penetration of the subsequent polymer reagent. Afterwards, it was exposed to a secondary antibody for 15 minutes, diaminobenzidine (DAB; Dako Real EnVision Detection System, Peroxidase/DAB +, Rb/Mo, Dako, Denmark) for staining reaction (as a chromogen), and Mayer’s haematoxylin for background staining. The sections were washed with TBS (tris-buffered saline) (pH, 7.4). Finally, the slides were dehydrated in graded alcohol, cleaned with xylene, and covered with a coverslip. The positive control for D2-40 and CD31 antibodies was a normal tonsil, while the negative control was obtained by omission of the primary antibody.

For immunohistochemical analysis of microvessel density (MVD), nuclear and cytoplasmic staining of vascular endothelial cells was evaluated with CD31. The cell units, cluster of cells, and vascular lumen formation without smooth muscles (stained and differentiated from tumor cells and adjacent mesenchymal tissues) were counted as a blood vessel (Eshghyar et al., 2008). On the other hand, to examine lymphatic vessel density (LVD), D2-40 reaction was evaluated by cytoplasmic staining of lymphatic endothelial cells. This positive reaction was evaluated by counting D2-40-stained lymphatic vessels, containing visible lumen which was clearly surrounded by lymphatic endothelial cells and other components of connective tissues (Kaur and Gupta, 2013).

MVD and LVD were determined using an Olympus optical microscope (BX41, Olympus,Tokyo, Japan). First, parts of histopathological slides from six regions (three intratumoral and three peritumoral regions) were selected with maximum MVD (hot spots) at 10× magnification; then, the number of blood vessels was counted in these regions at 40× magnification. After determining MVD in the intratumoral and peritumoral regions, the mean MVD was determined in intratumoral and peritumoral regions, and the mean number of blood vessels, stained with CD31 in each slide, was recorded (Taher et al., 2012).

Evaluation of LVD was conducted similar to MVD. First, parts of histopathological slides were selected from six regions (three intratumoral and three peritumoral regions) with maximum LVD (hot spots) at 10× magnification. Then, the number of blood vessels in each area was counted at 10× magnification. After determining LVD in the intratumoral and peritumoral regions, the mean LVD was measured in the intratumoral and peritumoral regions of the tumor (Taher et al., 2012). Moreover, D2-40 expression was evaluated in tumor cells. The immunoactivity of D2-40 in cytoplasmic and nuclear forms was considered as a positive reaction. The percentage of positive tumor cells was evaluated as follows (Taher et al., 2012): (-) < 10%; (+), 10-25%; (++), 26-50%; and (+++), 51-100%. 

MVD and LVD of tumors (adenoid cystic carcinoma and mucoepidermoid carcinoma), as well as other data, were analyzed using Chi square test, Pearson’s correlation test, t test, paired t test, and Spearman’s correlation test. In all tests, the significance level was set at 0.05.

## Results

In the present study, a total of 40 paraffinated blocks of malignant salivary gland tumors were evaluated, including 20 mucoepidermoid carcinoma and 20 adenoid cystic carcinoma blocks. A total of 20 mucoepidermoid carcinoma samples, including 11 (55%) samples with low-grade malignancy, seven (35%) samples with moderate-grade malignancy, and two (10%) samples with high-grade malignancy were included in the study; they were also confirmed in the histopathological evaluation. 

Out of 20 adenoid cystic carcinoma samples, 14 showed a cribriform pattern (70%), four showed a tubular pattern (20%), and two had a solid histopathological pattern (10%); the histopathological reevaluation confirmed these findings. The patients’ information (e.g., age and sex) is presented in Table 1. Pearson’s Chi square test was also used to compare the tumors.

In Table 2, intratumoral MVD, peritumoral MVD, total MVD, intratumoral LVD, peritumoral LVD, and total LVD of tumors are recorded. Comparison of tumors was conducted using t test.

Based on the paired t test, intratumoral and peritumoral MVD, as well as intratumoral and peritumoral LVD, were compared in mucoepidermoid carcinoma and adenoid cystic carcinoma (Table 3).

Based on the Pearson’s correlation test, the relationship between variables of age, intratumoral MVD, peritumoral MVD, total MVD, intratumoral LVD, and total LVD of tumors was evaluated. 

In mucoepidermoid carcinoma, age had no significant correlation with intratumoral or total MVD of the tumor. However, there was a significant positive correlation between age and peritumoral MVD, intratumoral LVD, peritumoral LVD, and total LVD (P= 0.024, P= 0.009, P= 0.038, and P= 0.10). There was also a significant positive correlation between intratumoral MVD and peritumoral MVD (P= 0.014), intratumoral MVD and total MVD (P< 0.001), intratumoral MVD and LVD (P= 0.019), and intratumoral MVD and total LVD of the tumor (P= 0.024). 

Nevertheless, there was no significant relationship between intratumoral MVD and peritumoral LVD. Similarly, there was no significant correlation between peritumoral MVD, intratumoral LVD, peritumoral LVD, and total LVD, while there was a positive relationship between peritumoral and total MVD of the tumor (P< 0.001). In addition, total MVD showed a significant positive correlation with intratumoral and total LVD (P= 0.017 and P= 0.024). On the other hand, total MVD had no significant relationship with peritumoral LVD, while there was a significant association between intratumoral and peritumoral LVD (P< 0.001). Also, total LVD had a significant relationship with intratumoral and peritumoral LVD (P< 0.001 and P< 0.001, respectively).

In adenoid cystic carcinoma, no significant relationship was found between age and other variables (MVD and LVD). Intratumoral MVD had a significant positive correlation with peritumoral and total MVD (P= 0.005 and P< 0.001, respectively). However, there was no significant correlation between intratumoral MVD, intratumoral LVD, peritumoral LVD, and total LVD in the tumor. Nonetheless, peritumoral MVD showed a significant relationship with total MVD, intratumoral and peritumoral LVD, and total LVD (P< 0.001, P= 0.043, P= 0.043, and P= 0.024, respectively).

**Figure 1:**
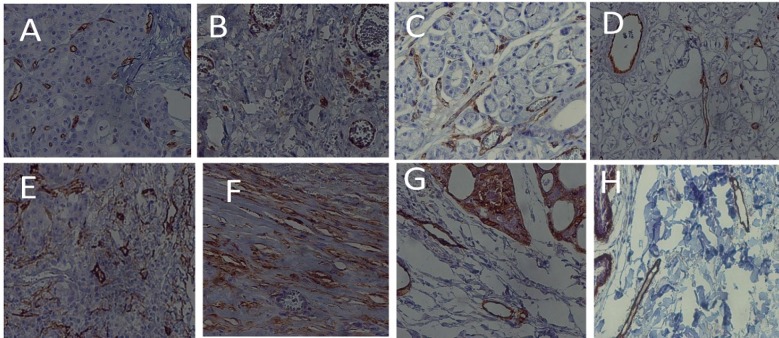
Staining of Blood Vessels with CD31 Marker in: A) mucoepidermoid carcinoma, intratumor (40×); B) mucoepidermoid carcinoma, peritumor (40×); C) adenoid cystic carcinoma, intratumor (40×); and D) adenoid cystic carcinoma, peritumor (40×). Staining of lymphatic vessels with D2-40 marker in: E) mucoepidermoid carcinoma, intratumor (40×); F) mucoepidermoid carcinoma, peritumor (40×); G) adenoid cystic carcinoma, intratumor (40×); and H) adenoid cystic carcinoma, peritumor (40×).

Furthermore, there was no significant relationship between total MVD and intratumoral and peritumoral LVD in adenoid cystic carcinoma. On the other hand, total LVD showed a significant correlation with peritumoral and intratumoral LVD (P< 0.001 and P<0.001, respectively). Also, there was a significant positive correlation between intratumoral and peritumoral LVD of the tumor (P< 0.001). In addition, distribution of tumor cell percentage, stained with D2-40 marker, was examined in mucoepidermoid carcinoma and adenoid cystic carcinoma (Table 4) (Figure 1).

**Figure 2:**
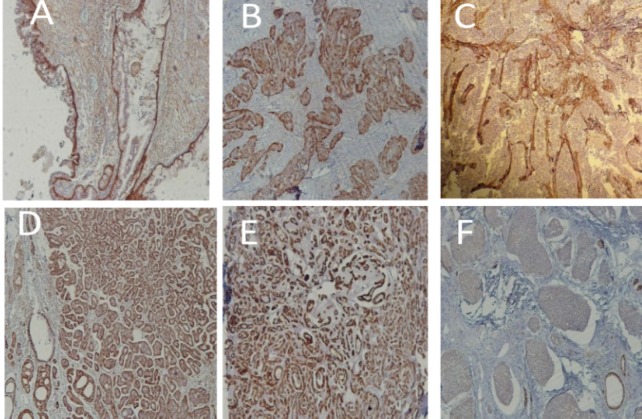
Staining of Tumor Cells with D2-40 Marker in Mucoepidermoid Carcinoma (A-C): A) low grade (10×); B) moderate grade (10×); C) high grade (10×). Staining of tumor cells with D2-40 marker in adenoid cystic carcinoma (D-F): D) cribriform pattern (10×); E) tubular pattern (10×), and F) solid pattern (10×).

Based on the results of Spearman’s correlation test, there was no significant relationship between age, malignancy grade, and D2-40-stained tumor cell percentage in mucoepidermoid carcinoma (P> 0.05). In the Spearman’s correlation test, there was also no significant relationship between age, histopathological subtype, and D2-40-stained tumor cell percentage in adenoid cystic carcinoma (P> 0.05). According to Spearman’s correlation test, histopathological grade had no significant relationship with total MVD, intratumoral MVD, peritumoral MVD, total LVD, intratumoral LVD, and peritumoral LVD in mucoepidermoid carcinoma and adenoid cystic carcinoma (P> 0.05) (Figure 2).

**Table 1:** The Frequency Distribution of Patients’ Clinical Information (age and sex)

Tumor/ Variables	Mucoepidermoid carcinoma	Adenoid cystic carcinoma	P value
Age	40.05±18.175	43.25±16.328	0.568
Sex			
Male	12	4	0.01
Female	8	16	

**Table 2:** Comparison of MVD and LVD between Mucoepidermoid Carcinoma and Adenoid Cystic Carcinoma

Tumor/ Mean±SD	Mucoepidermoid carcinoma	Adenoid cystic carcinoma	P value
Intratumoral MVD	49.92±20.676	15.18±9.629	< 0.001
Peritumoral MVD	27.20±15.155	18.83±15.111	0.089
Total MVD	34.81±16.233	17.01±11.134	< 0.001
Intratumoral LVD	11.43±6.524	5.87±6.668	0.011
Peritumoral LVD	5.65±2.175	8.03±15.370	0.496
Total LVD	8.61±4.079	7.23±10.925	0.6

**Table 3:** Comparison of Intratumoral and Peritumoral MVD and Intratumoral and Peritumoral LVD in Mucoepidermoid Carcinoma and Adenoid Cystic Carcinoma

Mean±SD/ Tumor		Intratumoral	Peritumoral	P value
Mucoepidermoid carcinoma	MVD	49.92±20.676	27.20±15.155	0.001
	LVD	11.43±6.524	5.65±2.175	< 0.001
Adenoid cystic carcinoma	MVD	15.18±9.629	18.83±15.111	0.193
	LVD	5.87±6.668	8.03±15.370	0.313

**Table 4:** Percentage Distribution of Tumor Cells Stained with D2-40 Marker

Percentage of tumor cells stained with D2-40		< 10%	11% - 25%	26% - 50%	> 50%	Total
Mucoepidermoid carcinoma	Number (Percent)	0 (0)	1 (5)	6 (30)	13 (65)	20 100
Adenoid cystic carcinoma	Number (Percent)	1 (5)	0 0	1 (5)	18 (90)	20 100
Percentage of total (MECs & ADCCs)		(2.5)	(2.5)	(17.5)	(77.5)	100

## Discussion

According to the results of the present study, total MVD in mucoepidermoid carcinoma was greater than total MVD in adenoid cystic carcinoma. In a study by Luukka et al. MVD in mucoepidermoid carcinoma was significantly different from MVD in adenoid cystic carcinoma (Luukkaa et al, 2009). Additionally, Tadbir et al. examined benign and malignant salivary gland tumors (i.e., pleomorphic adenoma, mucoepidermoid carcinoma, and adenoid cystic carcinoma) and reported the highest MVD in mucoepidermoid carcinoma (Tadbir et al, 2012). 

Dhanuthia et al., (2013) in agreement with the results reported by Tadbir et al., considered MVD as a distinguishing factor between malignant and benign salivary gland tumors. Glebernetto et al., (2012) also emphasized on the high degree of angiogenesis in mucoepidermoid carcinoma of minor salivary glands. However, in a study by Seify et al., (2014) there was no significant difference although MVD in mucoepidermoid carcinoma was greater than that of adenoid cystic carcinoma.

In the present study, the incidence of intratumoral MVD in mucoepidermoid carcinoma was higher than that of intratumoral MVD in adenoid cystic carcinoma. In the study by Seify et al., (2014) this difference was also noted, although it was not significant. The peritumoral MVD in mucoepidermoid carcinoma was greater than peritumoral MVD in adenoid cystic carcinoma; nevertheless, there was no significant difference. In the study by Seify et al., this difference was insignificant.

The higher MVD in mucoepidermoid carcinoma versus adenoid cystic carcinoma may explain the differences in the clinical behaviors and metastatic potential of tumors; also, center of tumor was found to play a more important role, compared to other regions. In other words, MVD had a more significant contribution to the clinical behaviors of mucoepidermoid carcinoma compared to adenoid cystic carcinoma, and intratumoral angiogenesis was more important than peritumoral angiogenesis. 

Total LVD was also higher in mucoepidermoid carcinoma, compared to adenoid cystic carcinoma; however, the difference was insignificant. In our literature review, we could not find any similar studies examining LVD in these two types of malignant salivary gland tumors; therefore, this study may be the first to address this phenomenon. Moreover, in the present study, intratumoral LVD was higher in mucoepidermoid carcinoma, compared to adenoid cystic carcinoma. Nonetheless, peritumoral LVD was not significantly different between tumors, although it was higher in adenoid cystic carcinoma.

In 60% of mucoepidermoid carcinoma samples, local and distant metastases were observed. Moreover, 44% of mucoepidermoid carcinomas of parotid glands (except low-grade lesions) metastasized to neck lymph nodes (Regezi et al., 2017). In adenoid cystic carcinoma, only 6% to 10% of carcinomas metastasized to lymph nodes, which is in fact uncommon (Neville et al, 2016). Considering the metastatic differences of tumors, the higher intratumoral LVD in mucoepidermoid carcinoma may be associated with metastasis to regional lymph nodes. Therefore, intratumoral LVD in these tumors may be considered a predictive factor for metastasis to regional lymph nodes.

In mucoepidermoid carcinoma, intratumoral MVD was higher than peritumoral MVD; however, Seify et al., (2014) did not find any significant differences. As reported in various cancers, intratumoral MVD is more significant than peritumoral MVD (Cardoso et al., 2009). According to a study by Bolzoni et al., (2009) intratumoral MVD in advanced oropharyngeal cancer was significantly higher than peritumoral MVD. Also, in a study by Safaei Naraghi et al., (2009) intratumoral MVD was higher than peritumoral MVD in basal-cell carcinoma.

On the other hand, in the present study, peritumoral MVD was higher than intratumoral MVD in adenoid cystic carcinoma; however, the difference was not significant. According to the results reported by Gasparini et al., (1995) peritumoral MVD was higher than intratumoral MVD in lung and breast cancers. Intratumoral MVD was found to be higher than peritumoral MVD in some tumors, while peritumoral MVD was greater than intratumoral MVD in others; it seems that biological behavior and type of tumor may be influential.

In the analysis of lymphangiogenesis in mucoepidermoid carcinoma, intratumoral LVD was higher than peritumoral LVD, and the difference was statistically significant. Nevertheless, in a study by Gleber-Netto et al., (2012) on mucoepidermoid carcinoma of minor salivary glands, peritumoral LVD was greater than intratumoral LVD, which is not consistent with the results of the present study. In our study, peritumoral LVD was higher than intratumoral LVD in adenoid cystic carcinoma; however, the difference was not significant. Fujita et al., (2011) and Starek et al., (2015) have also confirmed this finding and reported no significant difference.

In mucoepidermoid carcinoma, there was a significant relationship between total MVD and total LVD; however, there was no significant association in adenoid cystic carcinoma. In other words, as total MVD increases in mucoepidermoid carcinoma, total LVD improves, as well; accordingly, angiogenesis and lymphangiogenesis progress closely together in mucoepidermoid carcinoma. In these tumors, higher intratumoral versus peritumoral angiogenesis and lymphangiogenesis can be used as factors to justify clinical and metastatic behaviors of tumors. 

In this study, there was a significant relationship between age, total LVD, intratumoral LVD, and peritumoral LVD in mucoepidermoid carcinoma. In other words, intratumoral, peritumoral, and total LVD increased, as age at disease onset advanced. Based on this finding and the assumed relationship between lymphangiogenesis and metastasis to regional lymph nodes, it can be concluded that with advancing age, metastasis to regional lymph nodes increases in mucoepidermoid carcinoma. Nonetheless, there was no significant relationship between age, LVD, and MVD in adenoid cystic carcinoma. 

As the findings revealed, there was no significant relationship between the histopathological grade of tumor, total MVD, intratumoral MVD, and peritumoral MVD in mucoepidermoid carcinoma. Similarly, there was no significant relationship between histopathological grade and total LVD, intratumoral LVD, and peritumoral LVD. In a study by Taher et al., (2012) MVD and LVD showed no significant relationship with histopathological grade, which is in agreement with the results of the present study. Likewise, Seify et al., (2014) found no relationship between the degree of histopathological malignancy and MVD in mucoepidermoid carcinoma. On the other hand, Etemad Moghadam et al. reported a rare finding and showed a significant relationship between histopathological grade and MVD (Etemad Moghadam et al., 2010).

In adenoid cystic carcinoma, there was no significant relationship between the histopathological subtype and MVD or LVD. Similarly, Seify et al., (2014) did not find any correlation between MVD and histopathological subtype in adenoid cystic carcinoma. In mucoepidermoid carcinoma, there was no relationship between age, degree of malignancy, and percentage of tumor cells stained with D2-40; in 65% of these samples, more than 50% of cells were stained. Moreover, in adenoid cystic carcinoma, there was no relationship between age, histopathological subtype, and percentage of tumor cells stained with D2-40 marker; in 90% of these samples, more than 50% of tumor cells were stained. Since more than 50% of tumor cells were stained in 77.5% of all samples, this marker can be used as a potential therapeutic target; however, further analysis is necessary in a larger sample size.

A minor finding of this study was the significant difference between tumor groups in terms of gender. The prevalence of adenoid cystic carcinoma was higher among females. The Surveillance, Epidemiology, and End Results (SEER) program data in the United States (1973-2007) showed a male-to-female ratio of 1:1.4, which is consistent with the results of the present study, although the study populations were different (Neville et al., 2016). According to the present study, mucoepidermoid carcinoma was more prevalent among males. However, most studies have shown similar patterns in both genders (Neville et al., 2016, Gnepp, 2009). In this regard, Boukheris et al., (2009) which used SEER data from 1992 to 2006, showed that mucoepidermoid carcinoma was more prevalent in old men. Moreover, although the number of female patients was higher in adenoid cystic carcinoma, the prevalence decreased with aging.

In conclusion, the higher MVD in mucoepidermoid carcinoma compared to adenoid cystic carcinoma could justify the differences in clinical behaviors of tumors. Seemingly, the center of tumor played a more significant role in mucoepidermoid carcinoma, compared to other regions. Considering the differences in metastasis of tumors to regional lymph nodes, the higher intratumoral LVD in mucoepidermoid carcinoma might be related to metastasis to regional lymph nodes; the center of tumor also played a more significant role. As the findings revealed, MVD and LVD had no significant relationship with the patient’s age, histopathological grade, and percentage of tumor cells stained with D2-40 in mucoepidermoid carcinoma; a similar finding was also reported in adenoid cystic carcinoma.
